# Localization versus delocalization of *d*-states within the $\hbox {Ni}_{{2}}$MnGa Heusler alloy

**DOI:** 10.1038/s41598-022-23575-1

**Published:** 2022-11-29

**Authors:** Jozef Janovec, Martin Zelený, Oleg Heczko, Andrés Ayuela

**Affiliations:** 1 Donostia International Physics Center (DIPC), Manuel de Lardizabal 4, 20018 San Sebastián, Spain; 2 Centro de Física de Materiales-MPC CSIC-UPV/EHU, Manuel de Lardizabal 5, 20018 San Sebastián, Spain; 3 Faculty of Mechanical Engineering, Institute of Materials Science and Engineering, Brno University of Technology, Technická 2896/2, Brno, 61669 Czech Republic; 4 Faculty of Mathematics and Physics, Charles University, Ke Karlovu 5, Prague, 12116 Czech Republic; 5 FZU – Institute of Physics of the Czech Academy of Sciences, Na Slovance 1999/2, Prague, 18221 Czech Republic

**Keywords:** Electronic structure, Magnetic properties and materials

## Abstract

We present calculations based on density-functional theory with improved exchange-correlation approaches to investigate the electronic structure of $\hbox {Ni}_2$MnGa magnetic shape memory alloy prototype. We study the effects of hybrid functionals as well as a Hubbard-like correction parameter U on the structural, electronic and magnetic properties of the alloy. We show that the previously successful application of U on Mn should be extended by including U on Ni to describe the *d* localized electrons more accurately and in better agreement with experiments. The bonding interactions within this intermetallic alloy are analysed including the role of non-transition metal. We found that the strongest and most stabilizing bond is formed between the Ga–Ni pairs due to the delocalized *s*–*s* and *p*–*s* orbital hybridization. Our findings suggest that minimization of the over-delocalization error introduced by standard semi-local exchange-correlation functionals leads to a better description of the $\hbox {Ni}_2$MnGa alloy. Furthermore we propose that the experimental total magnetic moment of Ni–Mn–Ga alloys could be increased after carefully selected heat treatment procedures.

## Introduction

**Figure 1:**
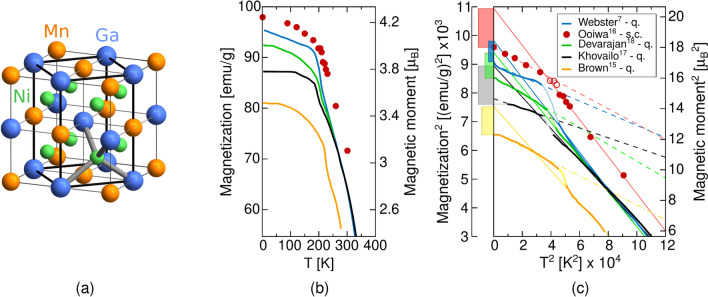
Depiction of (**a**) conventional L2$_1$ austenite unit cell with 8-atom computational cell highlighted, (**b**) experimentally measured magnetization saturation as a function of temperature, and (**c**) the same data represented in squared coordinates with extrapolation of linear sections to 0 K (solid line for austenite and dash line for martensite), following Ref.^16^. The legend includes abbreviations q. and s.c. to denote quenched and slowly cooled samples after the high temperature homogenization, respectively. The magnetization of austenite extrapolated to 0 K is higher than that of martensite (the difference is given by colour bars in the left axis).

Ferromagnetic alloys with the stoichiometry around $\hbox {Ni}_2$MnGa are well-known for their magneto-mechanical properties predominantly involving the magnetic shape memory (MSM) effect^1,2^, that is, a non-negligible deformation induced by an external magnetic field with attained maximum strains up to 12%^3^. The MSM occurs in a low-temperature martensite phase arising from the structural reorientation of highly mobile twin boundaries. This is caused by the alignment of the local magnetic moments with the external field due to the large magnetocrystalline anisotropy^1,2,4,5^. The high temperature austenite phase of $\hbox {Ni}_2$MnGa is characterized by the cubic $\hbox {L2}_{{1}}$ structure^6^, typical of Heusler alloys (see Fig. 1a). Upon cooling, the austenite phase undergoes one or multiple transformations into modulated martensite with $c/a < 1$ (periodic stacking of nanotwinned planes) and tetragonal non-modulated (NM) martensite with parallel crystallographic planes and $c/a > 1$, depending on stoichiometry^7–9^. The martensite stabilization of $\hbox {Ni}_{{2}}$MnGa is generally seen as a consequence of the interplay between nesting on the Fermi surface^10,11^, electron–phonon interactions^12,13^ and the Jahn–Teller effect^14,15^, closely related to Ni *d* orbitals.

In Ni–Mn–Ga alloys, ferromagnetism originates from Mn and Ni having unpaired valence electrons in their *d*-block, with the major contribution to the magnetization due to Mn atoms. Experimental values of the total magnetic moment vary between $\mu _\text {tot}$ = 3.4–4.2 $\mu _\text {B}$/f.u.^6,7,15–18^. A wide range of the measured magnetization saturation values (see Fig. 1b) originates from the high sensitivity of Ni–Mn–Ga alloys to composition. In particular, $\mu _\text {tot}$ decreases in both Mn deficient/excess off-stoichiometric alloys since the additional manganese atoms tend to occupy Ga sites where they align antiferromagnetically with the Mn sublattice^19^. Within near stoichiometric alloys, such disorder is present in the L2$_1$ structure as a trace of the phase transformation from the B2′ simple cubic structure consisting of one Ni-occupied and one Mn/Ga shared sublattice with complete disorder between Mn and Ga atoms. The B2′ to L2$_1$ ordering transformation temperature is approximately 800 $^{\circ }\hbox {C}$ for stoichiometric composition and occurs at even lower temperatures in off-stoichiometric alloys^20^. It is noteworthy that the disorder between Ga and Mn atoms occupying the same sublattices is found in most samples as a result of conventional heat treatments at high temperatures^7,15^.

The heat treatment of samples includes homogenization usually at 800 $^{\circ }\hbox {C}$ or higher followed by quenching^7,17,18^. The ordering transformation was reported to be almost instantaneous^20^ hence we expect no residual disorder between Mn and Ga at room temperature. Nevertheless, it was shown that the saturation magnetization can be increased by annealing^21^. After 18 h annealing at 315 $^{\circ }\hbox {C}$ the magnetic moment of the martensite phase increased by around 29% and of the L2$_1$ phase by about 1 $\mu _B$ compared to rapid quenched ribbon sample, linked to an increase in the Curie temperature. Such annealing procedure decreases the concentration of both Mn vacancies as well as Mn atoms on Ga sites that are antiferromagnetically coupled to the other ferromagnetic sublattices^19,22^ which effectively increases the total magnetic moment. Moreover, Fig. 1b,c show that higher magnetization can be measured on slowly cooled samples^16^ as opposed to the quenched ones. These facts suggest that Mn/Ga disorder is present even in the low temperature phases and more precise heat treatment leads to a measurement of larger magnetic moment than the values obtained after standard preparation procedures that nowadays include high homogenization temperatures and subsequent quenching.

Understanding the structural and magnetic couplings in Heusler alloys is key to the ongoing research, where first-principles calculations have become an invaluable tool^5^. Density-functional theory (DFT) based codes traditionally use the exchange-correlation potential described by the local density approximation (LDA)^23,24^ or the semi-local generalized gradient approximation (GGA)^14,25,26^. Despite their efficiency in many applications, LDA and GGA are well known to underestimate electronic band gaps^27^ and to poorly describe correlation of localized *d* and *f* electrons. This behaviour results from the over-delocalization of the electronic wave function caused by the self-interaction error inherent to DFT methods^28,29^. A better description of correlated systems can be obtained by applying hybrid functionals^30^ or by increasing the on-site Coulomb interaction within the DFT +U method inspired by the Hubbard model^31,32^.

Hybrid functionals are based on an admixture of the exact non-local Hartree–Fock (HF) and local or semi-local DFT exchange while the correlation functional is kept from DFT. A significant drawback of calculating the HF exchange is its slow decay in space, typical of long-range interactions, that results in higher computational time requirements. To overcome expensive integrations over the exchange interactions, Heyd, Scuseria and Ernzerhof proposed the screened hybrid functionals (HSE) in which the range of HF exchange is controlled by a tunable parameter^33^. In pure magnetic metals with direct *d*–*d* interactions, hybrid functionals were reported to overestimate magnetic moments^34–36^; however, Cu–Au alloys with fully occupied localized *d* shells, which are intertwined with *sp* bands, seem to be better described by hybrid functionals^37,38^. $\hbox {Ni}_2$MnGa is an intermetallic alloy composed of two transition metals with localized *d* orbitals hybridized with the broad *sp* Ga bands, which suggests more indirect interactions than in element bulk magnets. Experiments on Ni–Mn–Ga alloys with the modulated martensite phases show a pseudogap in [110] *k*-direction^39^ that already requires going beyond GGA to be explained^40^. Experiments using X-ray magnetic circular dichroism investigated nickel magnetic moments in more detail, and showed that the local Ni moments in Ni–Mn–Ga alloys behave differently from those in Ni bulk^41^. The Ni sites in $\hbox {Ni}_2$MnGa have also larger magnetic moments than 0.33 $\mu _B$^15–17^ assigned by using GGA. Then, all these results suggest performing further calculations with hybrid functionals for the case of Ni–Mn–Ga alloys.

Another approach that allows a better description of the localized orbitals is the Hubbard-like DFT +U model. Within this method, highly correlated *d* and *f* orbitals are selectively affected by an additional on-site Coulomb interaction while other electrons are treated by standard LDA or GGA approximation^42^. An advantage of DFT +U approach over the hybrid functionals is a considerably lower computational cost comparable with LDA or GGA. Previous DFT +U studies on Ni–Mn–Ga alloys used the U parameter only on Mn sites^43–46^ and they already reported improved description of the martensite phase giving better agreement with experimental data e.g. prediction of the correct ground state structure and improved lattice and elastic constants. The reported U values used on the Mn *d* orbitals range from U = 5.97 eV calculated by the linear response approach^43^ to U = 3.93 eV obtained by a fit to the experimental elastic constants^44^. Even a lower value of U = 1.8 eV was applied to Mn in works on doped Ni–Mn–Ga alloys in order to fit experiments on martensite phases^45,46^. The calculations with U on Mn larger than 1.2 eV correctly identify the five-layered modulated martensite (10M) as the ground state structure^47^ while GGA predicts a theoretical four-layered 4O martensite as the most stable structure^48^. On the other hand, calculations using GGA^49^ or U on Mn^46,47^ fail to predict the cubic phase as a high magnetic moment structure at 0 K. It follows from Fig. 1c that by extrapolating the linear part of the austenite magnetization curve to zero Kelvin we obtain higher magnetization of the austenite phase compared to the martensite phase. The literature review shows that U applied to Mn provides more accurate description of the modulated martensite structures, however, a comparison with the austenite phase is missing.

Even though Ni electrons are generally considered as delocalized and therefore U is omitted in calculations, the analysis of effective magnetic moments suggests that Ni subsystem has localized character^17^. Also, Ni has a significant effect on the energies and magnetic properties of the alloy^50^, therefore we need to study the U corrections not only on Mn but also on Ni atoms.

In this paper we consider two approaches to include the localized *d* electrons in the stoichiometric $\hbox {Ni}_2$MnGa alloy using the HSE03 hybrid functional and the DFT +U method. We first study trends for structural and magnetic properties based on the applied exchange-correlation method. Using several levels of theory we discuss the found tetragonal phases, and analyse their band structure as well as the chemical bonding.

## Results

### Structural, elastic and magnetic properties

**Figure 2:**
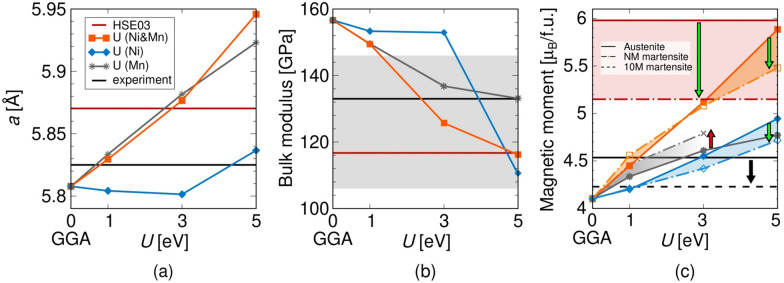
(**a**) Relaxed austenite lattice constant as a function of U applied to different sites compared with HSE03 and experimental value^6^, (**b**) bulk modulus calculated using HSE03 and DFT +U (colour lines) compared with experiments (black line^51^ and grey area^13,51,52^), and (**c**) total magnetic moments calculated using HSE03 and DFT +U compared with experimental value measured on modulated martensite^6^. Solid lines represent cubic austenite (in (**c**) panel the black solid line belongs to extrapolated value) and dash-dotted/dashed lines belong to NM/10M martensite. Both HSE03 and U on Ni predict larger total magnetic moment of austenite comparing to NM martensite at 0 K.

The relaxed equilibrium lattice constants of the austenite unit cell calculated with different methods are compared in Fig. 2a. Comparing several hybrid functionals, the austenite lattice constant using HSE03 is almost the same as when using HSE06^53^, and decreases by $0.01 {\text{\AA}}$ when calculated using the PBE0^54^. The unit cell volume of the austenite phase increases when the Hubbard U is applied to Mn atoms, when applied to Ni the volume first decreases slightly for U in the 1–3 eV range and then bounces back up at U = 5 eV.

We have next calculated the bulk modulus for the austenite phase by fitting the Murnaghan equation of state. The bulk modulus varies from 156 GPa predicted by GGA to 117 GPa obtained by HSE03, as shown in Fig. 2b. The available experimental values measured in the austenite phase at room temperature are valued around 133 GPa^51^. Note that the trend of having a lower austenite bulk modulus due to enhanced localization is in good agreement with experiments^13,51,52^. The sudden change in the bulk modulus versus U dependence at U (Ni) = 5 eV results from the use of a harder Ni potential including 3*p* valence electrons. Further test calculations with a softer Ni potential showed smooth trends for the bulk modulus and the lattice constant in the whole range of tested U values.

An increase in the magnetization calculated by HSE03 was expected due to stronger electron localization and pronounced exchange splitting, as observed in bulk metallic systems^35,36^. Calculations using meta-GGA exchange-correlation functional also predict higher magnetization than GGA^47,55^ (see also Supplementary Information SI Figs. 1, 2). Our HSE03 calculations predict the austenite total magnetic moment equal to 5.98 $\mu _\text {B}$/f.u. in contrast with 4.11 $\mu _\text {B}$/f.u. obtained by uncorrected GGA. The comparison of local magnetic moments using HSE03 and GGA shows that the Mn magnetic moment becomes larger by around 20%, and the Ni local magnetic moment is more than doubled when hybrid functionals are used. The total magnetization of the austenite phase calculated with other hybrid functionals is in agreement: the HSE06 and PBE0 calculations predict 5.91 $\mu _\text {B}$ and 5.78 $\mu _\text {B}$ respectively. These test calculations in the case of $\hbox {Ni}_2$MnGa alloy showed that the results obtained by the above hybrid functionals are similar, therefore calculations presented in the following are performed using the HSE03 functional because its convergence behaviour was found to be better for this system.

In general, the DFT +U calculations enhance the localization of transition metal *d* electrons hence we can see a growth of local magnetic moments when applying the U parameter on either Ni or Mn atoms. The total magnetic moment of the austenite phase calculated with HSE03 is closely approached when using U = 5 eV on Ni and Mn simultaneously. The total magnetization of the austenite and martensite phases is given as function of the U parameter in Fig. 2c. Note that the total magnetization of the austenite at 0 K is smaller than the one of the martensite when we use uncorrected GGA or U correction on Mn. Contrarily meta-GGA SCAN^47,55–57^, HSE03 and U on Ni all predict the austenite phase to have larger $\mu _\text {tot}$ at zero Kelvin (emphasized by arrows in Fig. 2c and SI Fig. 2c).

### Tetragonal distortion

**Figure 3:**
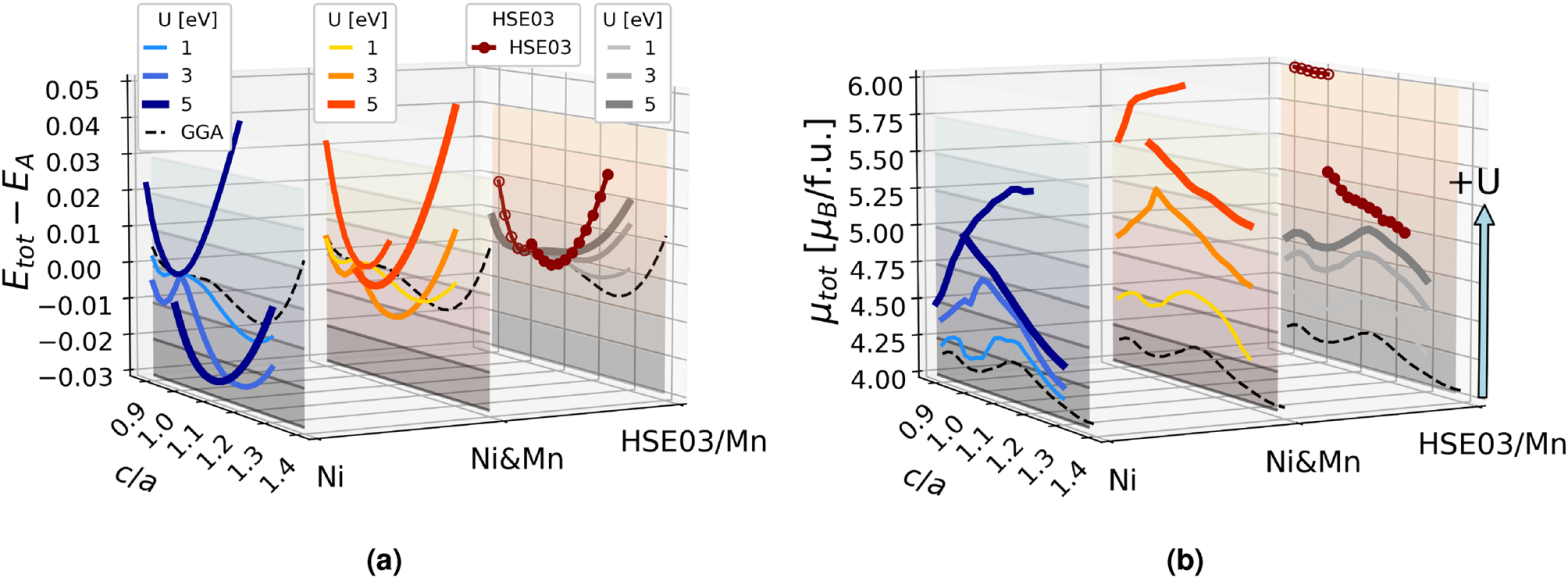
Comparison of (**a**) total energies, and (**b**) corresponding total magnetic moments per formula unit $\mu _{tot}$ (plotted on the *z* axis) along the tetragonal deformation path (*c*/*a* on *x* axis), calculated using HSE03 and DFT +U (U on Ni in blue, U on Ni and Mn in orange, U on Mn in grey). Standard GGA results are represented by black dashed lines. Energies are plotted relative to the energy of the austenite phase. 2D representation of Fig. 3 can be found in the Supplementary Information SI Fig. 1.

We investigated the stability of non-modulated (NM) tetragonal structures following the total energy as a function of the tetragonal deformation obtained by elongating and compressing the austenite phase^49^, while keeping the unit cell volume constant and equal to the equilibrium austenite volume to avoid non-systematic errors. We find that the energy versus *c*/*a* curve calculated with HSE03 has two distinct minima corresponding to two different magnetic states. Firstly, there is a minimum at the cubic ratio $c/a = 1$ that is characterized by a larger magnetic moment with value around 6 $\mu _\text {B}\text {/f.u.}$ (empty red circles in Fig. 3). Secondly, there is a martensite structure solution with $c/a \approx 1.08$, having the total magnetic moment around 5.1 $\mu _\text {B}\text {/f.u.}$ (full red circles in Fig. 3). Note that uncorrected GGA calculations indicated by the black dashed lines in Fig. 3 predict a global minimum of NM martensite at $c/a \approx 1.25$^49^. Compared with standard GGA results, the *c*/*a* ratio of the NM martensite calculated with HSE03 functional decreases, the corresponding energy minimum is shallow, which implies that its stability with respect to the austenite phase is lower. Furthermore, the slope of the energy curve versus *c*/*a* becomes steeper in the case of hybrid functionals, a fact that points towards a higher tetragonal shear modulus $C'$ predicted by hybrids.

When we use the U correction parameter simultaneously on Ni and Mn sites there is a combination of effects that arise when U is applied separately to Ni and Mn. On the one hand, U on Mn destabilizes the NM martensite^43^, and rapidly decreases its tetragonality (grey lines in Fig. 3a. On the other hand, using U on Ni strongly stabilizes the NM martensite and the *c*/*a* ratio decreases less rapidly. Furthermore, there is a new metastable tetragonal structure with $c/a < 1$ (blue lines in Fig. 3a) having *c*/*a* about 0.95 which is close to the experimental tetragonality of modulated martensite structures. It is noteworthy that GGA calculations predicted phases with $c/a < 1$ when using large computational supercells and the modulation of crystallographic planes^58,59^. The calculations with U on both Ni and Mn sites follow the same trends as in the case of U on Ni, but with less stabilized tetragonal structures due to the U parameter on Mn. The effect of U on Mn becomes more important in the case of U (Ni &Mn) = 5 eV because the NM martensite stability decreases, and the energy difference $\Delta \hbox {E}_{Austenite-NM}$ becomes comparable to the one calculated with HSE03. Concerning the *c*/*a* values of the NM martensite and the total magnetic moment, the best match between HSE03 and DFT +U method can be found in the range of U (Ni &Mn) = 3–5 eV. From the above analysis of tetragonal deformation we show that the structural stability of $\hbox {Ni}_2$MnGa alloy is strongly dependent on electron interactions involving Ni atoms, so that a more precise treatment of Ni *d* electrons should be carefully considered in theoretical calculations.

Figure 3 shows that in the case of HSE03 and U = 5 eV on Ni the *E* vs. *c*/*a* curve splits into the high magnetic moment cubic structure and the low magnetic moment tetragonal martensite. In order to investigate the dependence of crystal structure on magnetization, we perform fixed spin magnetic moment calculations along the tetragonal deformation path that indicate a strong influence of total magnetic moment on the present minima. Firstly, calculations using the HSE03 hybrid functional with fixed $\mu _\text {tot}$ = 4.1 $\mu _\text {B}\text {/f.u.}$ predict a martensite phase with *c*/*a* equal to 1.24, that is the tetragonality given by the result of the GGA calculations. Secondly, using GGA and imposing a net magnetic moment of 5.5 $\mu _\text {B}\text {/f.u.}$ leads to a *E* vs. *c*/*a* curve that resembles the tetragonal deformation path obtained by the HSE03 hybrid functional with a minimum at the tetragonality ratio *c*/*a* = 1.06. The same behaviour was found for DFT +U calculations with U = 3 eV applied simultaneously on Ni and Mn. These tests suggest that the magnetic moment becomes the leading parameter when analysing the stability of the $\hbox {Ni}_2\text{MnGa}$MnGa martensite structures, assumption that is supported by the experimental evidence since the paramagnetic austenite in off-stoichiometric alloys always transforms into the NM martensite^5^.

### Electronic structure

In order to understand effects of exchange-correlation potentials on the electronic structure we calculated band structures with different methods which can be found in the supplementary information material (SI Fig. 3). The GGA band structure shows broad bonding bands below − 4 eV and antibonding above the Fermi level ($E_F$), with a *s* and *s*–*p* like contribution of Ga states in these regions. In between we find the *d* bands belonging to Ni and Mn atoms that are flat and more localized. These interacting Ni *d* and Mn *d* orbitals show a distinct separation between bonding and antibonding states at the gamma point below $E_F$. The antibonding Mn *d* states in the spin-down channel are shifted above $E_F$ due to the strong Mn exchange splitting. The tetragonal splitting of the Ni states is mostly related to two $e_g$ orbitals that form an antibonding peak at $E_F$, which is held responsible for stabilizing the tetragonal phases by breaking its twofold degeneracy^14^.

**Figure 4:**
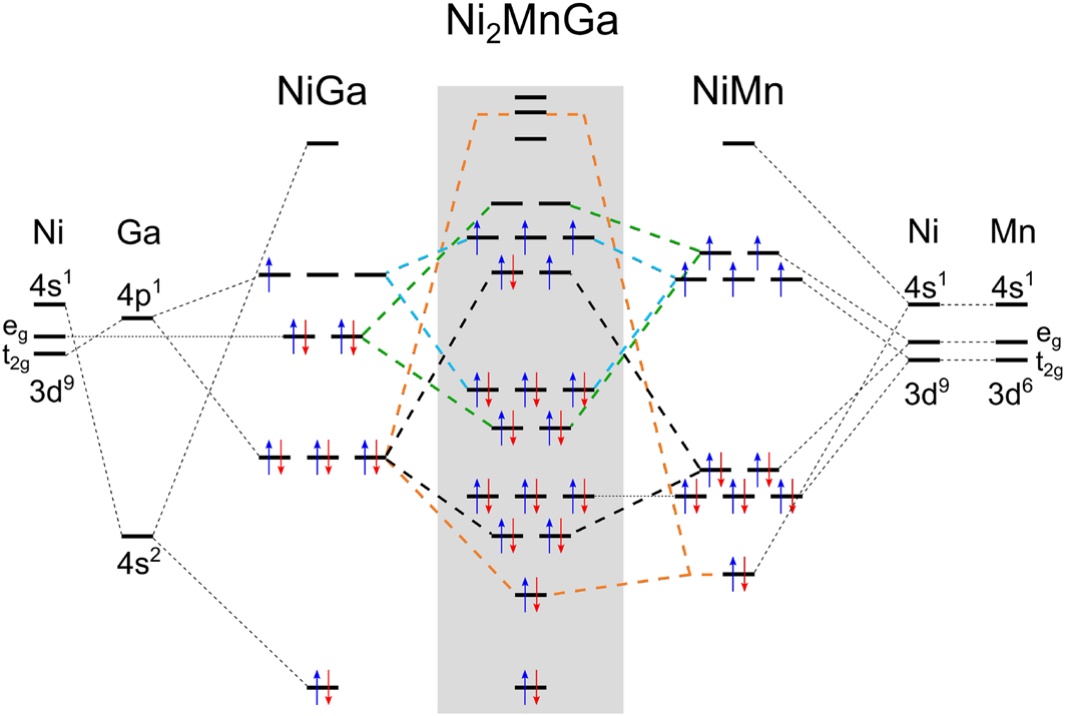
Orbital diagram for $\hbox {Ni}_2$MnGa. The main interaction between Ni and Ga lattices after hybridization is given on the left side; the intermediate interaction between Ni and Mn atoms on the right side. Subsequently the Ni–Ga levels are refined because of the Ni–Mn interaction to form the diagram for the $\hbox {Ni}_2$MnGa alloy. Both spin channels are depicted as degenerate for simplicity.

The hybrid functional (see SI Fig. 3) stabilizes all the spin-up bands as a result of a robust exchange splitting of Mn spin-down bands and to some extend of the Ni *d* electrons. The spin-down channels are nearly free of Mn states resulting in the Mn magnetic moment increase when compared to the GGA calculations. The spin-up valence region near $E_F$ consists of Ga *p* and Ni *d* hybridized orbitals. The band structure calculated with U = 5 eV on Ni and Mn reproduces the band separation of the HSE03 limiting case with an even stronger localization of the Ni states.

The flat spin-up Ni $e_g$ bands in the austenite phase are predicted by GGA to be positioned at $E_F$ which corresponds to the density of states (DOS) peak at the Fermi level and causes the instability of the cubic phase^14,15^. However, the same DOS peak calculated by HSE03 and DFT +U with large values of U shifts slightly above the Fermi level, and the splitting of this peak along the martensitic tetragonal deformation provides less stabilization than in the case of GGA. The energy difference between the austenite and the NM martensite is therefore considerably smaller in HSE03 and DFT +U with U = 5 eV on Ni and Mn than in standard GGA results, as Fig. 3a shows.

In GGA results the two uppermost spin-down orbitals of the austenite are shifted towards higher energies in a way that the half-filled spin-down $e_g^*$ orbital is positioned at $E_F$. The occupation of spin-up orbitals leads to an excess of 4 spin-up electrons corresponding to a magnetization around 4 $\mu _\text {B}\text {/f.u.}$ obtained by GGA. When we apply hybrid functionals or high U parameter, the exchange splitting increases and the energy of the spin-up orbitals decreases, so that the half-filled spin-down orbital moves above $E_F$. Thus, the number of spin-up electrons increases by one and the total magnetic moment amounts to 6 $\mu _\text {B}\text {/f.u.}$, which is close to values obtained using hybrids or high values of U.

### Bonding analysis

**Figure 5:**
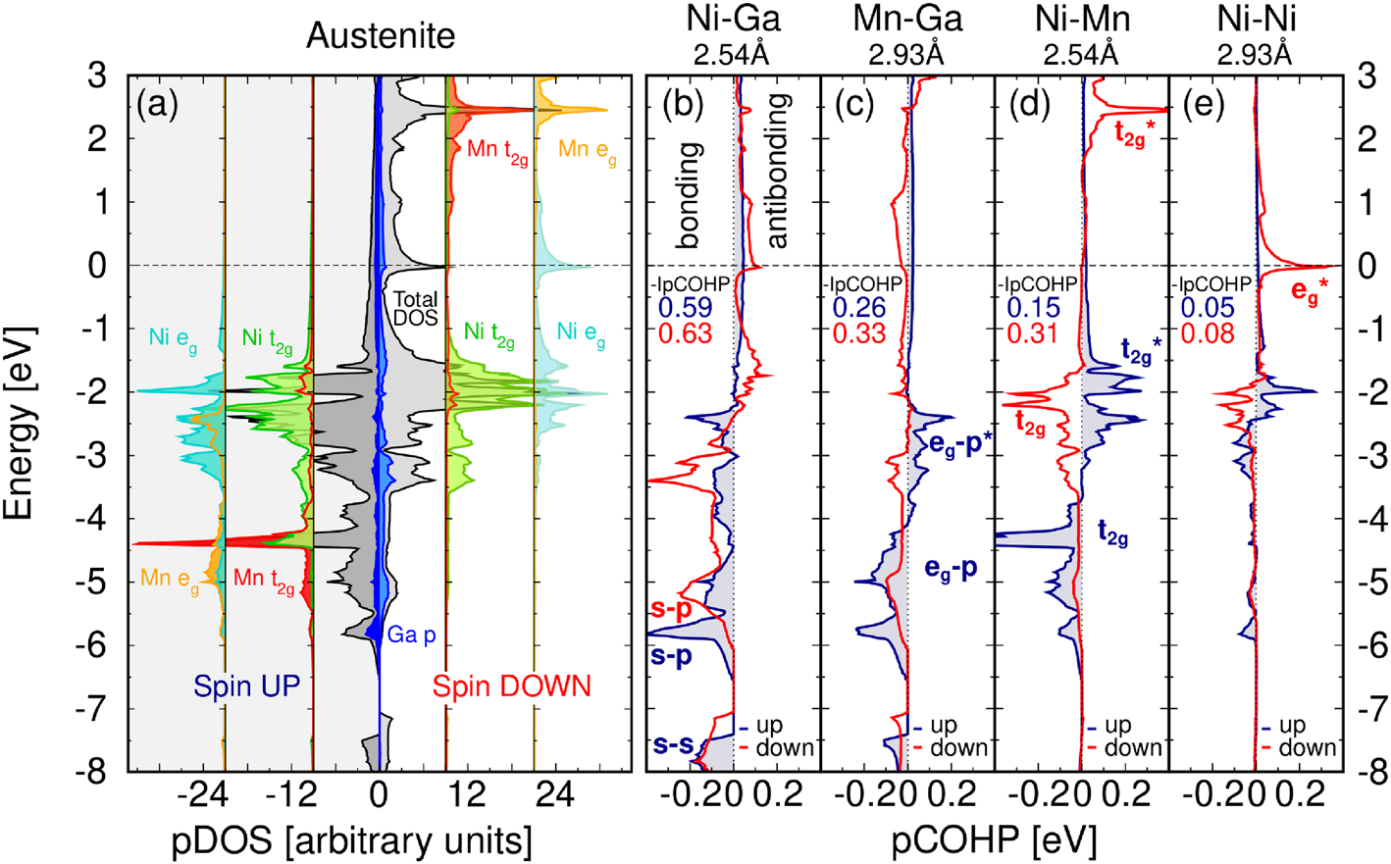
LOBSTER results of (**a**) pDOS projected on atoms, and (**b–e**) projected crystal orbital Hamilton population (pCOHP) calculated for atom pairs. The negative pCOHP represents stabilizing (bonding) while positive pCOHP represent destabilizing (antibonding) interactions, latter denoted by asterisk. The integrated values of IpCOHP/bond represent bond strength.

Besides the band structure calculations, in order to build an orbital diagram as shown in Fig. 4, we projected the optimized plane wave description of the problem onto a local basis set using the LOBSTER code^60,61^. The local basis set allows us to decompose the site DOS (abbreviated as pDOS) more accurately by incorporating part of the density that is outside of the projection spheres used by the VASP code. From the projected local orbitals we computed the projected crystal orbital Hamilton population (pCOHP)^62^, a bond analysis tool that details bonding and antibonding interactions. We focus mainly on the spin-up channel since the spin-down channel possesses similar features shifted due to the exchange splitting. The spin-up valence region calculated with U = 3 eV on Ni and Mn (Fig. 5) can be roughly divided into four regions: (i) within the range of energies between − 10 and − 7 eV the *s* region dominated by Ga electrons is shown, (ii) around − 6 eV there is the $s{-}p$ region with mainly Ga *p* and Ni *s* hybridization, (iii) between − 5.5 and − 2.5 eV the $p{-}d$ region is found where Ga *p* states mix with Ni *d* and Mn *d* states, and (iv) the *d* states dominate the region composed of Ni and Mn overlapping states in the interval from − 3 eV to $E_F$. Based on this picture we propose a possible orbital diagram for the $\hbox {Ni}_2$MnGa alloy (Fig. 4). The current alloy can be treated as a pseudobinary systems (Ni–Ga) (Ni–Mn) where each half is dealing with sublattices showing the major interactions.

The L2$_1$ structure can be seen as four interpenetrating FCC sublattices two of which are occupied by Ni atoms but not all sublattice interactions are equivalent. Focusing on the nearest neighbours we first consider the main interaction of Ni and Ga, then the secondary interaction between Ni and Mn. The orbitalwise projected pCOHP (see SI Fig. 4) identifies a significant contribution of Ni $t_{2g}$ orbitals in all the interactions with Ga *p* orbitals. We therefore first find the interaction of the Ni and Ga *s* orbitals dominating the low lying bonding region and next the Ni $t_{2g}$-Ga *p* interaction as the left part of Fig. 4 shows. Besides, the *d* orbitals of the Ni and Mn sublattices interact to form bonding and antibonding orbitals (the right part of Fig. 4). The sublattices interact through these two intermediate interactions. The low energy Ni–Ga *s* orbital is not affected and the *s* orbital due to the Ni–Mn interaction hybridizes with Ga *p* creating the bonding spin-up DOS peak at − 6 eV and shifted spin-down peak at − 5 eV (see pCOHP analysis in Fig. 5). The rest of Ga *p* orbitals interact with the bonding $e_g$ orbitals created by the Ni–Mn interaction, resulting in the spin-up bonding peak at − 5 eV and the antibonding peak around − 3 eV dominated by Mn–Ga interaction. The remaining bonding Ni–Mn $t_{2g}$ orbital forms peaks around − 4.5 eV in the spin-up channel. Last, the Ni *d* orbitals interact with the antibonding Ni–Mn *d* orbitals and they occupy higher lying energies.

The pCOHP curves are collected for the four shortest bonds in Fig. 5b–e, where the states are separated into their bonding and antibonding contributions, calculated using U = 3 eV on Ni and Mn. The total strength of a bond can be assessed by the value of pCOHP integrated up to $\hbox {E}_F$, namely IpCOHP, which gives the energy contribution of an individual bond to the band energy (IpCOHP values are included in Fig. 5). Based on the IpCOHP analysis, the strongest single bonds are formed between Ga and both transition metals. The strong *s*–*s* and *s*–*p* interaction makes the Ni–Ga bond almost twice as stable as those of Mn–Ga and Ni–Mn. The interaction of Mn with Ga is stronger than the Ni–Mn bond which is even shorter. This finding can be explained by the magnetic splitting that moves the *s*–*s* and *s*–*p* character antibonding orbitals in the Mn–Ga interaction above $E_F$. The *d*–*d* splitting in the Ni–Mn interaction is weaker, and the occupied spin-up $t_{2g} ^*$ antibonding states are weakening this bond. The spin-down Ni peak at the Fermi level that contributes to the austenite phase instability is a half-filled antibonding orbital due to Ni *d*-Ni *d* and Ni *d*-Ga *p* interactions (Fig. 5b,e) in agreement with theory^14^ and experiment^63,64^.

## Discussion

Calculations using GGA and hybrid functionals act as two limits in a description of *d* electrons, while DFT +U can be seen as an intermediate approach that is closer to one of the two limits depending on the chosen value of the U parameter. Hybrid functionals are likely to predict too large local magnetic moments on Ni sites in the austenite phase. The magnetic moment of Ni varies from 0.36 $\mu _\text {B}\text {/atom}$ by GGA to 0.97 $\mu _\text {B}\text {/atom}$ by HSE03 and 0.81 $\mu _\text {B}\text {/atom}$ by DFT +U with U = 5 eV on Ni. On the experimental side, the Ni magnetic moments are reported with values larger than 0.4 $\mu _\text {B}$/atom at cryogenic temperatures^15–17^ and the extrapolation to the 0 K assigns 0.56 $\mu _\text {B}$/atom to Ni in the austenite phase^16^.

We now bring our results on magnetic moments into close contact with experiments. Both hybrid functionals and DFT +U calculations point towards an increase of magnetic moment in comparison with GGA due to a stronger localization of *d* electrons. The currently available measurements on $\hbox {Ni}_{{2}}$MnGa show considerable differences in total magnetic moments arising from variations in composition and sample preparation. An aspect with a major impact on the measured magnetic moment is the atomic disorder between Mn and Ga sites, which is regularly introduced by heat treatments, being nowadays inherent in the protocols to obtain good shape memory properties. An increase of magnetization was reported in the samples that were slowly cooled from the homogenization temperature^16,65^. It follows that lower homogenization temperatures below the B2’ to L2$_1$ ordering temperature should be considered to retain the L2$_1$ ordered structure and after the annealing samples should be slowly cooled in order to allow for Mn-vacancy recombination. The magnetization extrapolation in Fig. 1c also suggests that a modification of the heat treatment process can increase the Curie temperature^65^.

Furthermore, experimental measurements under high magnetic fields show that the slope of the magnetization versus temperature curve of the austenite is steeper than that of the martensite as Fig. 1b,c shows. Therefore the magnetization obtained by extrapolating the austenite curve to 0 K is higher than the measured magnetization of the martensite. From experiments it can be estimated that the magnetic moment of the austenite at 0 K would be greater than 4.5 $\mu _\text {B}$/f.u.^16^. In addition, a measurement in the austenite phase above the Curie temperature has reported a localized paramagnetic moment of Mn around 3.85 $\mu _\text {B}$^7^. This means that the total magnetic moment of austenite at 0 K would be reliably estimated by our calculations as they predict a total magnetic moment around 5 $\mu _\text {B}$/f.u.

Based on the discussion above, the calculations should be able to predict both higher total magnetic moment as well as the larger local magnetic moment of Ni in the austenite when compared to the martensite at 0 K. Previous results using the U correction to GGA with U restricted to Mn atoms predicted higher magnetization of martensite phases. On the other hand, our HSE03 and DFT +U calculations with U on Ni predict a higher magnetization of austenite in agreement with the experimental trend. Because hybrid functionals produce large local magnetic moment on Ni, reasonable results are expected using intermediate values for the U parameter (U = 3 eV) on the Ni and Mn sites in Ni–Mn–Ga alloys.

The U parameter previously determined by seeking an agreement with the experimental bulk modulus equal to 133 GPa^51^ proposed U = 3.93 eV for the Mn sites^44^. By studying the calculated bulk modulus, we find that the agreement to the same experimental value (solid black line in Fig. 2b) is achieved with U = 5 eV on Mn only or U = 2–3 eV on both Ni and Mn (the difference arises from including the inner *p*-Ni electrons as valence in our calculations). It seems that in order to get a better agreement with the experimental bulk modulus using lower and more reasonable U values, we have to take into account the U correction also on Ni sites.

We further discuss the results obtained using a promising approach to exchange-correlation such as the recent functional meta-GGA SCAN^56^. SCAN results for Ni_2_MnGa are included in SI. Compared with GGA results, the austenite lattice constant is smaller, the magnetic moment is larger, and the *c/a* ratio of NM martensite is well reproduced^47,55,57^. Since the U parameter overestimates the lattice constant of austenite (see Fig. 2a), the use of meta-GGA SCAN +U could result in a better agreement with the experiments. Anyway, SCAN already approaches higher magnetic moments than GGA^47,55,57^ and correctly predicts higher magnetization of austenite over martensite phase in agreement with our results. Therefore, smaller values of the parameter U would be necessary in the meta-GGA + U approach, which is consistent with the metallic nature of the constituent elements.

Moreover, DFT +U plays a role in the stability of modulated martensite phases. It was reported that the U correction applied to Mn *d* orbitals already predicts the experimental 10 M martensite as the ground state structure^47^. The U parameter assigned to Ni favours tetragonal symmetry and even predicts structures with $c/a < 1$. We therefore show that U on Ni can be expected to stabilize modulated structures with tetragonality below *c*/*a* = 1 as well, however, this question needs further investigation employing a supercell approach including shuffling of planes that is beyond the scope of this work.

Last but not least, we suggest a simple approximation to take into account the effect of temperature using the fixed spin magnetic moment calculations, idea that was successfully implemented before^66^. We showed that the structural stability along the tetragonal deformation path is mainly guided by the total magnetic moment in all levels of approximation. The austenite phase corresponds to a high magnetic moment and the NM martensite to a low magnetization solution. We can see this splitting in HSE03 results as well as GGA +U (Fig. 4) and SCAN +U (see SI Fig. 1) with large values of U on Ni and Mn. By fixing larger total magnetic moment in the calculation we can destabilize the tetragonal structure and at certain point the high temperature cubic austenite becomes the stable structure. We thus have a simple approach to mimic temperature effects by increasing or decreasing the total magnetic moment per unit cell.

In summary, we studied structural, magnetic and electronic properties of $\hbox {Ni}_2$MnGa magnetic shape memory alloy using the HSE03 hybrid functional and the DFT +U approach. The hybrid functionals seem to improve the simulation accuracy as the magnetic moment increases due to localization on Ni atoms; however, it comes related with a large local magnetic moment and a small bulk modulus for the austenite phase. Thus, a highly advantageous approximation to the exchange-correlation functional for Ni–Mn–Ga alloys is the DFT +U method, which can serve as a bridge between delocalized GGA and localized hybrids by adjusting the level of localization with the U parameter. The use of the U parameter on Ni correctly predicts a higher magnetization of the austenite phase compared to the martensite at 0 K that is in agreement with experiments and has a physical origin. Furthermore, the NM martensite is stabilized, an effect that is missing when applying the U parameter is restricted to Mn. As a side effect, it predicts the existence of the tetragonal variant with *c*/*a* ratio lower than one, in agreement with experiments. It also helps to obtain a bulk modulus comparable with the available measured data with lower values of U than in the case of U solely on Mn. We therefore conclude that the U parameter around 3 eV should be assigned to both Mn and Ni in order to increase the accuracy of future DFT calculations on Ni–Mn–Ga alloys. In addition, there is a relation between the total magnetic moment and the found structural phases.

We have also performed a chemical bond analysis and showed that the interaction of Ga with Ni (and Mn) lowers the total energy more (is more stabilizing) than the interaction between transition metals. This suggests that Z elements in $\hbox {X}_2$YZ Heusler alloys play an important role in bonding interactions that contribute to the lowering of the total energy, a fact to be emphasized as Z elements are non magnetic and as such were not much commented in previous works.

## Methods

We used density functional theory in combination with projected-augmented wave potentials (PAW)^67^ as implemented in the Vienna ab-initio Simulation Package (VASP)^68,69^. The exchange-correlation potential following the hybrid functional HSE03^33^ is approximated by the full Perdew–Burke–Ernzerhof (PBE)^26^ correlation and a weighted combination of one-quarter of HF and three-quarters of PBE for the exchange part. The calculations using GGA and DFT +U^70^ were performed using the PBE parameterized exchange-correlation functional with non-spherical contributions inside the PAW spheres. The first Brillouin zone (BZ) of the eight atoms unit cell (shown in Fig. 1a) was sampled using the gamma-centered Monkhorst-Pack grids of $11 \times 11\times 5$ k-points in the case of the hybrids and $17 \times 17 \times 9$ in the case of DFT +U, k-meshes were tested for convergence. The Gaussian smearing method for integration over the BZ was used with a 0.05 eV smearing width. The plane wave electronic orbitals were expanded with a cut-off energy of 450 eV in HSE03 and 500 eV in DFT +U. For higher accuracy, valence electrons were set to Ni 3$\hbox {p}^{6}$ 3$\hbox {d}^{9}$ 4$\hbox {s}^{1}$, Mn 3$\hbox {p}^{6}$ 3$\hbox {d}^{6}$ 4$\hbox {s}^{1}$ and Ga 3$\hbox {d}^{10}$ 4$\hbox {s}^{2}$ 4$\hbox {p}^{1}$ in all calculations, including hybrid functionals. The total energy convergence criterion was set to 10$^{-4}$ eV and 10$^{-5}$ eV per unit cell for HSE03 and DFT +U, respectively.

We also performed the localized basis set calculations using the LOBSTER package^60,61^ that allowed us to study the chemical bonding in solid state materials. In the calculation setup, BZ integration uses the tetrahedron method with Blöchl corrections on a $15 \times 15 \times 11$ k-points grid, a 600 eV cut-off energy, and an electronic convergence criterion equal to 10$^{-6}$ eV per unit cell.

## Supplementary Information

Supplementary Information.

## Data Availability

The data obtained for purposes of this work can be requested from the corresponding author.
