# Publisher Correction: Localization versus delocalization of *d*-states within the Ni_2_MnGa Heusler alloy

**DOI:** 10.1038/s41598-023-29103-z

**Published:** 2023-02-09

**Authors:** Jozef Janovec, Martin Zelený, Oleg Heczko, Andrés Ayuela

**Affiliations:** 1 Donostia International Physics Center (DIPC), Manuel de Lardizabal 4, 20018 San Sebastián, Spain; 2 Centro de Física de Materiales-MPC CSIC-UPV/EHU, Manuel de Lardizabal 5, 20018 San Sebastián, Spain; 3 Faculty of Mechanical Engineering, Institute of Materials Science and Engineering, Brno University of Technology, Technická 2896/2, Brno, 61669 Czech Republic; 4 Faculty of Mathematics and Physics, Charles University, Ke Karlovu 5, Prague, 12116 Czech Republic; 5 FZU – Institute of Physics of the Czech Academy of Sciences, Na Slovance 1999/2, Prague, 18221 Czech Republic

Correction to: *Scientific Reports* 10.1038/s41598-022-23575-1, published online 29 November 2022

In the original version of the Article, Figure 2 was a duplication of Figure 1.

**Figure 1:**
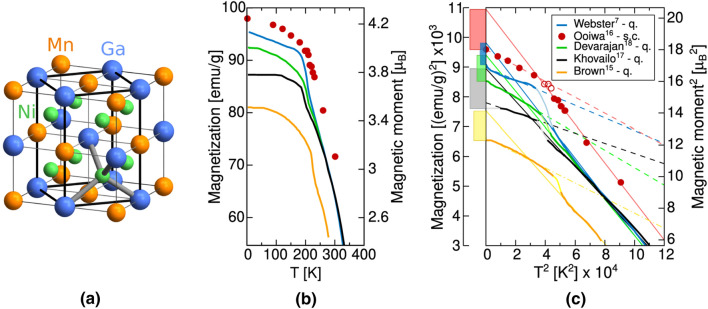
Depiction of (**a**) conventional L2_1_ austenite unit cell with 8-atom computational cell highlighted, (**b**) experimentally measured magnetization saturation as a function of temperature, and (**c**) the same data represented in squared coordinates with extrapolation of linear sections to 0 K (solid line for austenite and dash line for martensite), following Ref.^16^. The legend includes abbreviations q. and s.c. to denote quenched and slowly cooled samples after the high temperature homogenization, respectively. The magnetization of austenite extrapolated to 0 K is higher than that of martensite (the difference is given by colour bars in the left axis).

**Figure 2:**
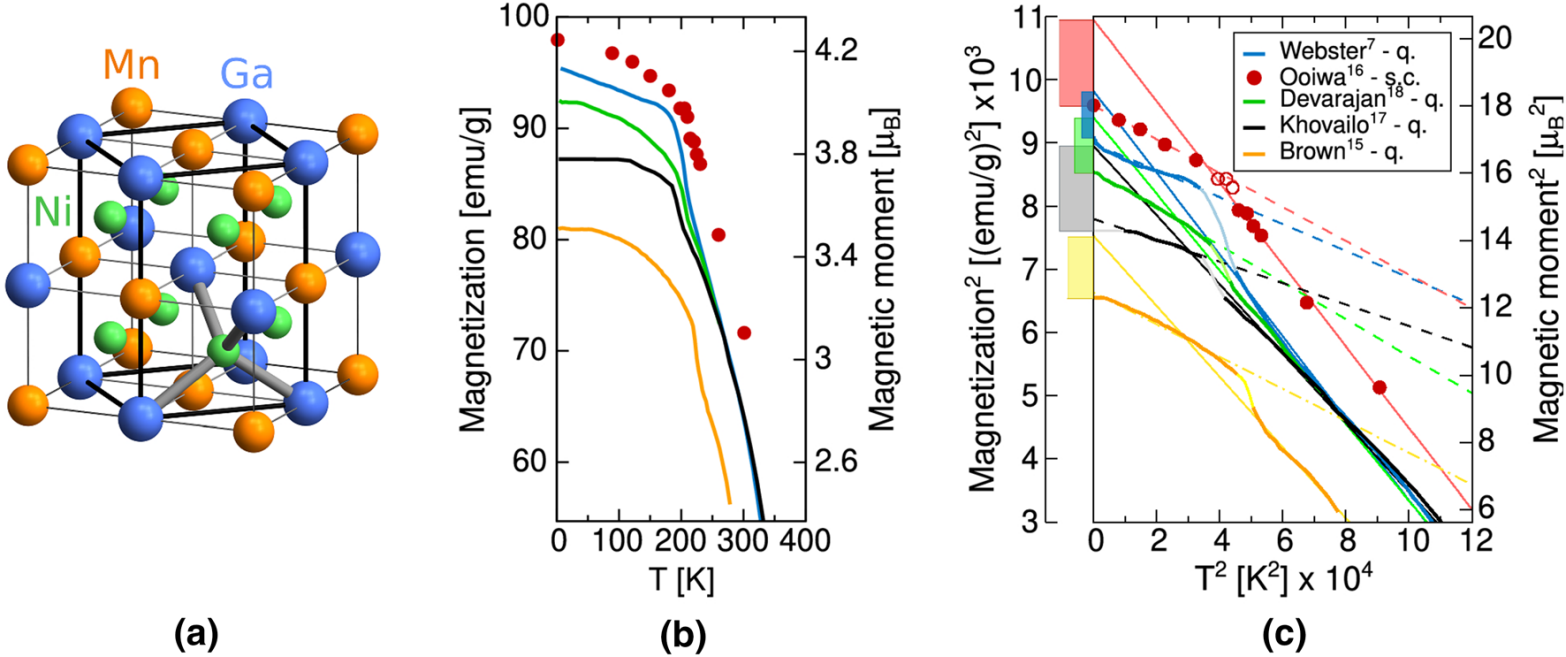
(**a**) Relaxed austenite lattice constant as a function of U applied to different sites compared with HSE03 and experimental value^6^, (**b**) bulk modulus calculated using HSE03 and DFT + U (colour lines) compared with experiments (black line^51^ and grey area^13,51,52^), and (**c**) total magnetic moments calculated using HSE03 and DFT + U compared with experimental value measured on modulated martensite^6^. Solid lines represent cubic austenite (in (**c**) panel the black solid line belongs to extrapolated value) and dash-dotted/dashed lines belong to NM/10 M martensite. Both HSE03 and U on Ni predict larger total magnetic moment of austenite comparing to NM martensite at 0 K.

In addition, in Figure 1, panel (c), the label of the Y-axis ‘Magnetization^2^ [(emu/g)^2^] × 10^3^’ was incorrectly given as ‘Magnetization [(emu/g)^2^] × 10^3^’.

The original Article has been corrected.

