# Supplementary material for: Localization versus delocalization of d-states within the Ni2MnGa Heusler alloy
Source: Sci Rep. 2022 Nov 29;12:20577. doi: 10.1038/s41598-022-23575-1 (PMC9708842; doi:10.1038/s41598-022-23575-1)
Supplement: Supplementary file 1 — Supplementary Information. [file 41598_2022_23575_MOESM1_ESM.pdf]

Supplementary Information

Localization versus Delocalization of d-States within the Ni_2MnGa Heusler Alloy

Jozef Janovec, Martin Zelený, Oleg Heczko, Andrés Ayuela*

*a.ayuela@csic.es

Supplementary material 1 - Tetragonal distortion and SCAN +U calculations

As an alternative to the GGA exchange-correlation in combination with the U correction parameter, we tested the meta-GGA SCAN +U approach. The SCAN calculation set up was the same as used for GGA +U calculations described in the methods section, using the first-order Methfessel-Paxton method with Gaussian broadening of 0.1 eV to integrate the Brillouin zone.

The SCAN calculations with U on Ni and Mn (green curves in SI Figure 1(d) and (h)) predict the same trends for the studied properties as GGA +U. Calculated lattice constants are smaller and the magnetic moments are larger, even exceeding the HSE03 value already at U = 3 eV. The lattice constant calculated with SCAN +U = 1 eV agrees well with the experimental lattice parameter of the austentie extrapolated to 0K (long-dashed black line in SI Figure 2(a)) using the linear expansion coefficient[1]. The SCAN +U correctly predicts higher magnetization of the austenite compared to the martensite in the whole range of U values. The magnetic moment predicted by HSE03 is reached at U = 3 eV on Ni and Mn. Furthermore, obtaining the high magnetization austenite and the lower magnetization martensite curves separately is reproduced. Hence, SCAN calculations also predict that the magnetization is the leading parameter concerning the stability of tetragonal structures in Ni_2MnGa. The calculated values of the analysed properties are summarized in Table 1.

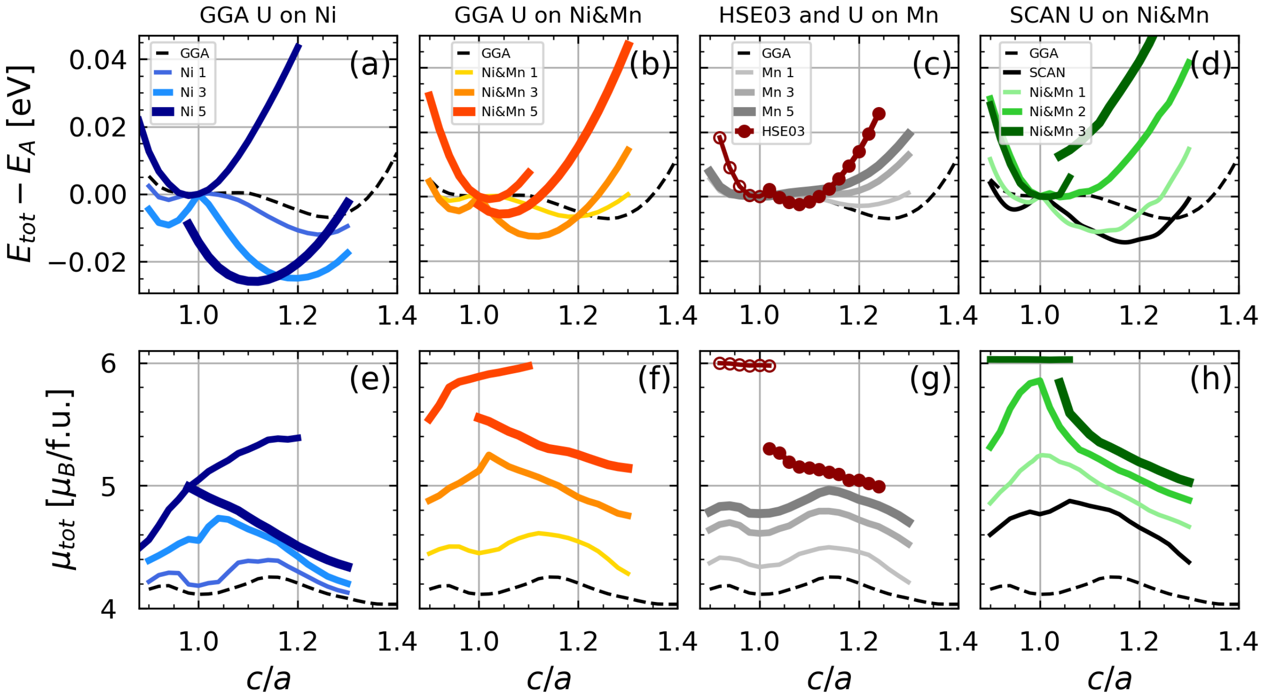

SI Figure 1. Comparison of structural and magnetic properties as a function of tetragonality c/a calculated using three levels of approximation - GGA, SCAN and hybrid functional HSE03. The three upper panels show the total energy as a function of tetragonality. Numbers specified in legend boxes represent numerical values of the U parameter in eV used on preceding element or combination of elements. Lower row of panels contains corresponding magnetic moments per formula unit. This graph is a 2D representation of Figure 3 in the main body of the paper, now extended by SCAN and SCAN +U with U on Ni and Mn simultaneously.

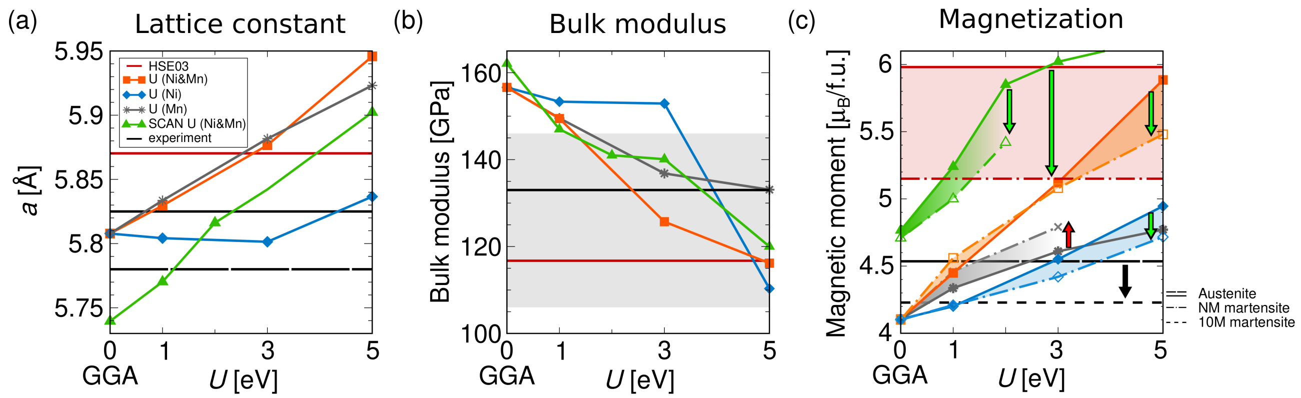

SI Figure 2. Results obtained using the U parameter in combination with GGA and meta-GGA SCAN (green lines) compared with HSE03 hybrid functional: (a) lattice constant of the austenite compared with high temperature experimental data[2] (solid black line) and extrapolation to 0K using the linear expansion coefficient[1] (long-dashed black line), (b) bulk modulus compared with experiment (grey area[3–5]), and (c) magnetic moment per formula unit related to measured magnetic moment of 10M martensite (dashed black line) and the extrapolated magnetic moment of austenite at 0K[6] (long-dashed black line).

SI Table 1. Calculated lattice parameters a_A, bulk modulus and magnetic moments for the cubic austenite, tetragonality of calculated structures and magnetic moments for the NM martensite. Experimental values for the austenite[2–5] and the 10M martensite phase[6,7] are also included.

Method		AUSTENITE					NM MARTENSITE				
		a_A [Å]	B [GPa]	μ_{tot} [μ_B/f.u.]	μ_{Mn}	μ_{Ni}	$c/a > 1$	$c/a < 1$	μ_{tot} [μ_B/f.u.]	μ_{Mn}	μ_{Ni}
HSE03		5.870	117	5.98	4.12	0.97	1.079	-	5.15	4.06	0.67
U (NiMn)	U = 1 eV	5.830	149	4.45	3.69	0.40	1.188	0.943	4.61	3.68	0.49
	U = 3 eV	5.877	126	5.12	4.09	0.54	1.113	0.957	5.07	4.07	0.55
	U = 5 eV	5.946	116	5.89	4.33	0.83	1.046	-	5.48	4.32	0.66
U (Ni)	U = 1 eV	5.804	153	4.20	3.41	0.41	1.245	0.937	4.21	3.32	0.49
	U = 3 eV	5.801	153	4.55	3.47	0.57	1.193	0.936	4.42	3.35	0.60
	U = 5 eV	5.846	108	4.95	3.47	0.81	1.111	0.981	4.74	3.42	0.76
U (Mn)	U = 1 eV	5.834	150	4.34	3.67	0.35	1.203	-	4.46	3.64	0.43
	U = 3 eV	5.882	137	4.61	4.07	0.28	1.130	-	4.79	4.07	0.37
	U = 5 eV	5.923	133	4.77	4.32	0.23	-	-	-	-	-
SCAN		5.740	162	4.77	3.69	0.55	1.171	0.948	4.73	3.68	0.49
U (NiMn)	U = 1 eV	5.770	147	5.24	3.92	0.68	1.116	0.976	5.02	3.88	0.62
	U = 2 eV	5.816	142	5.85	4.11	0.89	1.047	-	5.44	4.08	0.75
	U = 3 eV	5.832	140	6.03	4.22	0.96	-	-	-	-	-
GGA		5.808	157	4.11	3.41	0.36	1.261	-	4.10	3.32	0.42
Experiment		5.825*	106-153*	4.53**				0.939	4.23		

*room temperature measurement

**extrapolated to 0 K

Supplementary material 2 - Electronic band structure

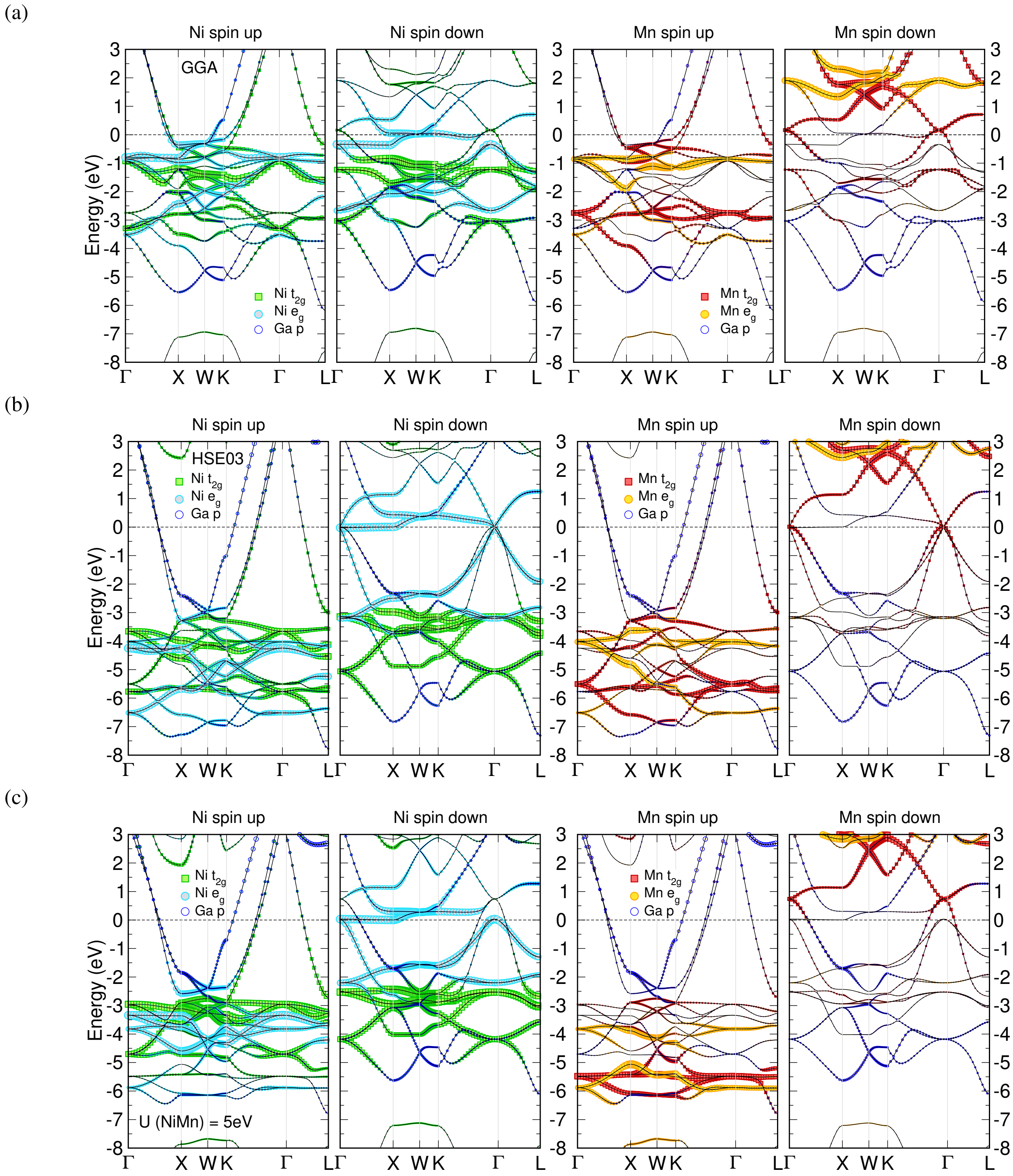

SI Figure 3. Electronic band structure of austenite for both spin channels calculated with (a) GGA, (b) HSE03, (c) DFT +U (GGA +U) with U = 5 eV on Ni and Mn. The projected t_{2g} and e_g orbitals of Ni and Mn alongside with Ga p contribution are highlighted.

Supplementary material 3 - Bond analysis

The pCOHP analysis of the four bonds projected on single orbitals obtained using the LOBSTER package as shown in SI Figure 4 - SI Figure 7. The interaction between Ni t_{2g} and Ga p is in SI Figure 4 divided into the in-plane (dark blue) and out of-plane (cyan) alignment, schematically shown in the inset.

(a)

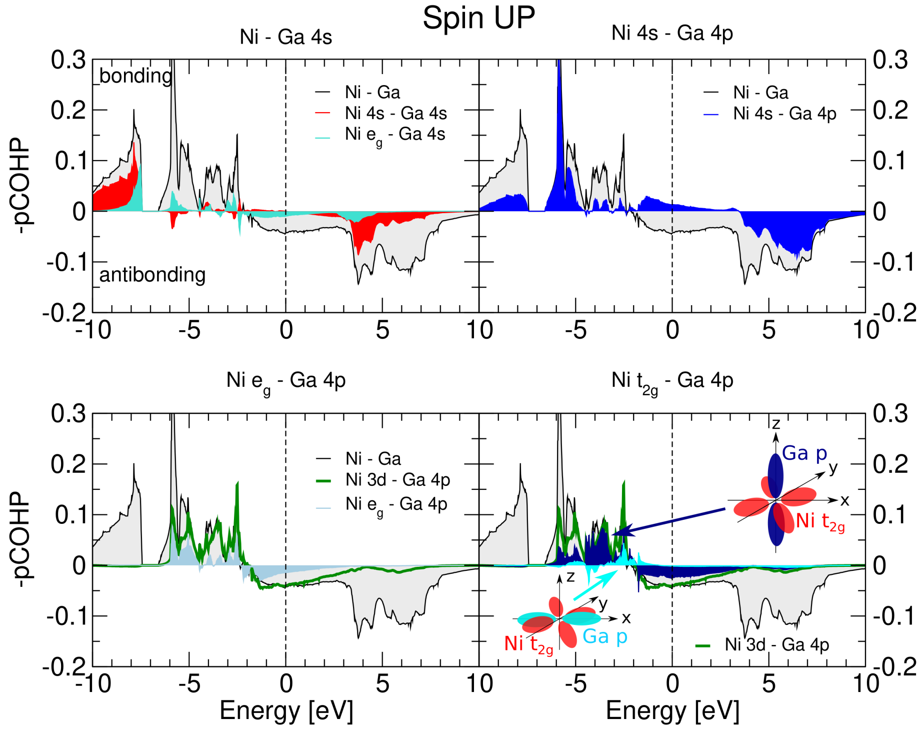

(b)

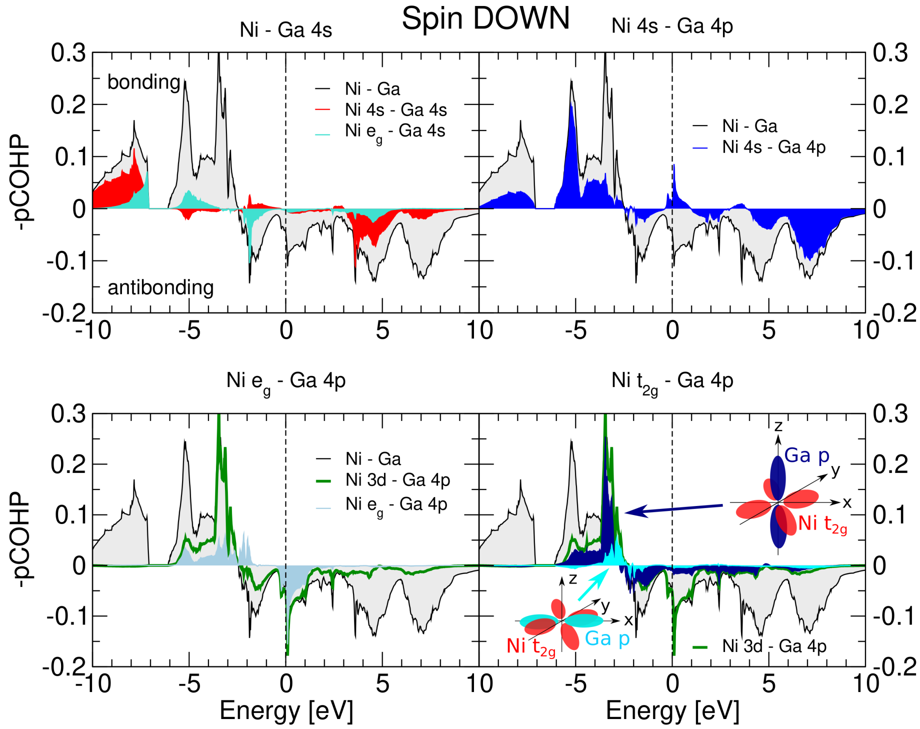

SI Figure 4. The pCOHP projected on orbitals for the strongest Ni - Ga interaction in (a) spin up and (b) spin down channel.

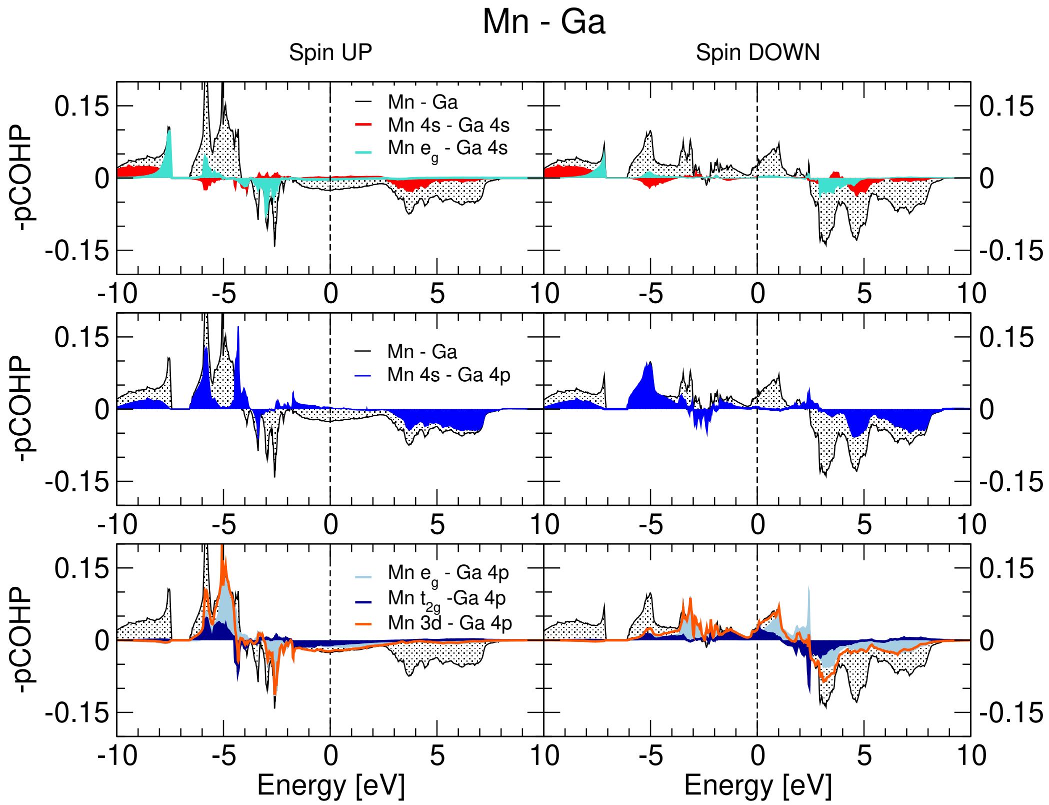

SI Figure 5. The pCOHP projected on orbitals of the Mn - Ga interaction in spin up and spin down channel.

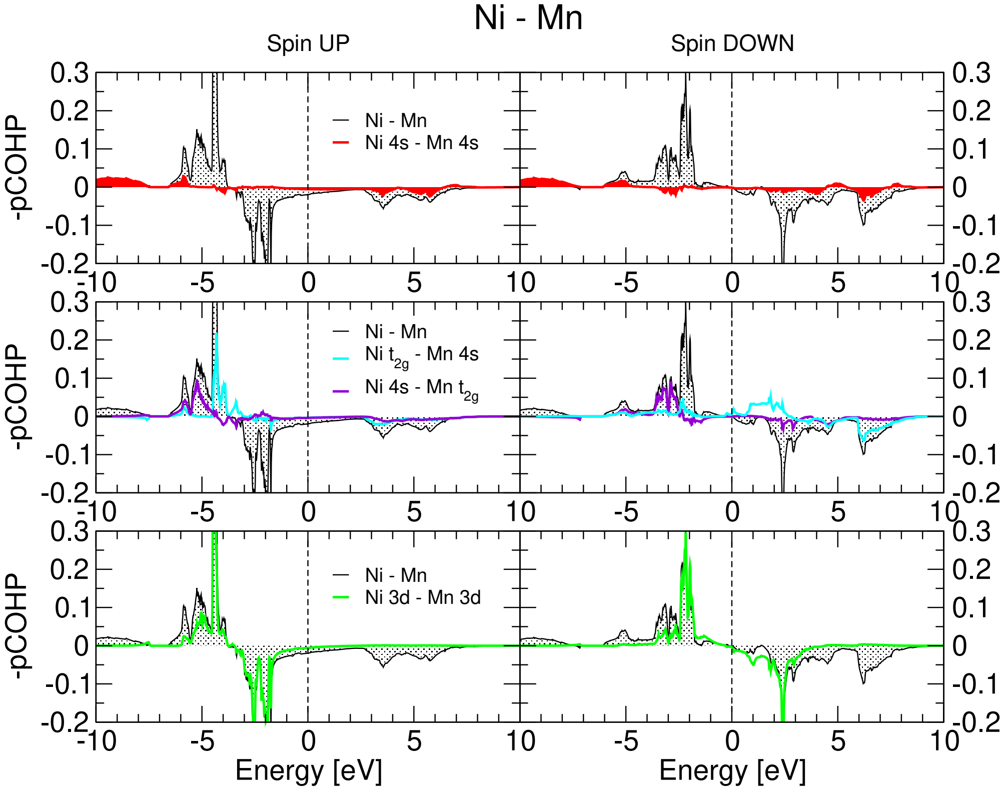

SI Figure 6. The orbital projected pCOHP for the Ni - Mn bond in spin up and spin down channels.

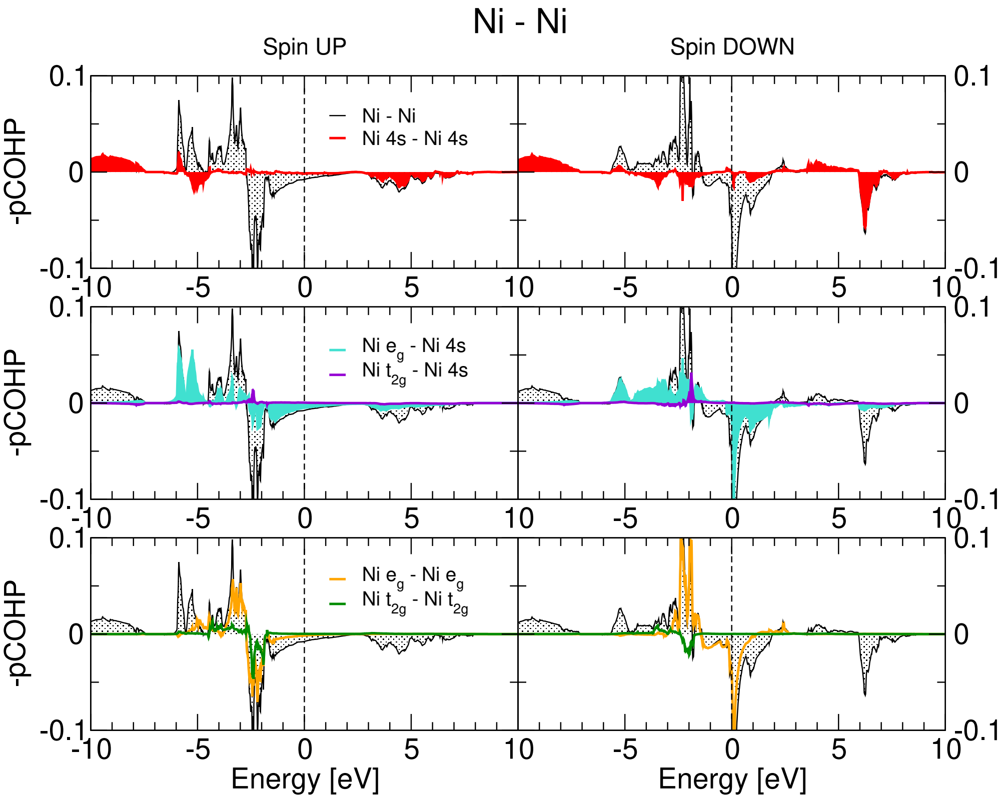

SI Figure 7. The orbital projected pCOHP for the Ni - Ni interaction in spin up and spin down channels.

References

1. Buchelnikov, V. D. *et al.* The thermal expansion coefficient and volume magnetostriction of Heusler Ni_2MnGa alloys. *J. Magn. Magn. Mater.* **300**, e459–e461 (2006).
2. Webster, P. J. Heusler alloys. *Contemp. Phys.* **10**, 559–577 (1969).
3. Worgull, J., Petti, E. & Trivisonno, J. Behavior of the elastic properties near an intermediate phase transition in Ni_2MnGa. *Phys. Rev. B* **54**, 15695 (1996).
4. Seiner, H. *et al.* Combined effect of structural softening and magneto-elastic coupling on elastic coefficients of NiMnGa austenite. *J. Alloy Compd.* **577**, S131–S135 (2013).
5. Mañosa, L. *et al.* Anomalies related to the TA_2-phonon-mode condensation in the Heusler Ni_2MnGa alloy. *Phys. Rev. B* **55**, 11068 (1997).
6. Ooiwa, K., Endo, K. & Shinogi, A. A structural phase transition and magnetic properties in a Heusler alloy Ni_2MnGa. *J. Magn. Magn. Mater.* **104**, 2011–2012 (1992).
7. Martynov, V. V. & Kokorin, V. V. The crystal structure of thermally- and stress- induced martensites in Ni_2MnGa single crystals. *J. Phys. III* **2**, 739–749 (1992).
